# Using LLMs to Infer Non-Binary COVID-19 Sentiments of Chinese Microbloggers

**DOI:** 10.3390/e27030290

**Published:** 2025-03-11

**Authors:** Jerry Chongyi Hu, Mohammed Shahid Modi, Boleslaw K. Szymanski

**Affiliations:** Department of Computer Science and Network Science and Technology Center, Rensselaer Polytechnic Institute, Troy, NY 12180, USA; huc3@rpi.edu (J.C.H.); modim2@rpi.edu (M.S.M.)

**Keywords:** sentiment analysis, Weibo, COVID-19, social media

## Abstract

Studying public sentiment during crises is crucial for understanding how opinions and sentiments shift, resulting in polarized societies. We study Weibo, the most popular microblogging site in China, using posts made during the outbreak of the COVID-19 crisis. The study period includes the pre-COVID-19 stage, the outbreak stage, and the early stage of epidemic prevention. We use Llama 3 8B, a large language model, to analyze users’ sentiments on the platform by classifying them into positive, negative, sarcastic, and neutral categories. Analyzing sentiment shifts on Weibo provides insights into how social events and government actions influence public opinion. This study contributes to understanding the dynamics of social sentiments during health crises, fulfilling a gap in sentiment analysis for Chinese platforms. By examining these dynamics, we aim to offer valuable perspectives on digital communication’s role in shaping society’s responses during unprecedented global challenges.

## 1. Introduction

The COVID-19 pandemic was one of the most disruptive events in recent history, and its effect on social media was profound. It caused widespread panic and led to the proliferation of misinformation and fake news through media outlets, the internet, and social networks [1]. It caused measurable differences in the sentiments and emotions expressed by social media users through their posts, including on the Chinese microblogging website Weibo [2].

In this paper, we analyze user sentiments on the Weibo platform during the initial five-month period during which the pandemic started to spread in China (November 2019–March 2020). We categorize millions of Chinese posts into four sentiment categories (positive, negative, neutral, and sarcastic) using the Meta Llama 3 8B Instruction-tuned variant LLM [3]. Then, we analyze them to describe the sentiment dynamics on Weibo. This LLM is pre-trained on a 15T multi-language token corpus [4]. As language understanding tasks are an important goal for the Llama 3 herd of models and many modern inference LLMs in general, our task is an interesting use case of LLMs at scale. The 8B variant of the Llama 3 herd of models can run on a single 80GB VRAM GPU, representing a good compromise between model size and reasoning power.

While popular social media such as Facebook reported removing large numbers of posts and users to prevent the spread of misinformation and fake accounts during the pandemic [5], it is unclear to what extent Chinese social media Weibo moderated posts during this period. Weibo’s content moderation system is community-driven and has several unique features. For instance, some Weibo users can voluntarily report posts that promote unsocial behavior. The platform treats such reports differently, and a panel of community jurors is sometimes selected from the platform’s users to make case decisions instead of relying just on platform administration [6].

Thus, it is essential to consider how moderation and censorship may have affected users’ present sentiments, as users may have self-censored or masked sensitive posts to avoid community reporters or AI-based detection. One way they may have done this is by using sarcasm; we measure sarcastic posts independently of their positive or negative sentiments. Conceptually, we treat sarcasm as a separate sentiment category, such that a post categorized as sarcastic conveys a sarcastic tone instead of plain positivity or negativity. Sarcastic posts may sometimes represent a negative or positive message, but the deliberate use of sarcasm supersedes positive or negative sentiment classification for such posts. This work’s novel contributions are (1) a technique for large-scale Chinese text sentiment classification using an LLM instead of standard NLP techniques and (2) sentiment dynamics analysis of pandemic-time Weibo posts.

The following section reviews some literature about Weibo, sentiment analysis, and LLM-based classification. Next, we describe our Methods, including our dataset and few-shot prompting approach. We then present our results and discuss possibilities for future work.

### Related Work

Studies, like [1], have been conducted about the impact of misinformation during the COVID-19 pandemic. These studies show that social media, mainstream media, and other sources of information inadvertently propagate misinformation or false information. This misinformation likely contributes to increased negative or sarcastic tones in social media response posts.

Opinion mining can reveal shifts in societal sentiments during crises, especially when users have polarized views among affected communities, including in response to government policies. A study on the sentiments of Turkish and English language tweets about the Syrian refugee crisis [7] found differences in crisis sentiments and perspectives between communities based on their language. Similar studies on press articles and social media posts about the European refugee crisis [8] and the United Airlines crisis [9] also showed that sentiment trends about crises are reflected in discussions on online platforms, providing a unique window into emerging beliefs. The shifting sentiments of Weibo users to COVID-19-related public health policies were also studied in [10], showing that public reactions gradually increased in negativity after initial positive support.

A study on perceptions of government legitimacy and how they affected individual willingness to disclose private information to the government in Shanghai, China [11] found that moral legitimacy took precedence over cognitive legitimacy, with altruism being the mediator. The study states that Chinese moral and collective values played an important role in privacy disclosures, and this finding may help explain sentiment dynamics on Weibo. In addition, ref. [12] showed that Weibo users used memes to avoid online censorship. Such use of memes supports our decision to track sarcasm as a sentiment/tone, as Weibo users may have attempted to avoid moderation.

In [13], the authors examined 700,000 Twitter posts and 400,000 Weibo posts over three months from December 2020 to February 2021. The study compared sentiments about COVID-19 vaccines on Twitter and Weibo and found that neutral posts were most common on Twitter. At the same time, positive tweets were most common on Weibo, among other key observations. In contrast, our study examines the broader sentiment on Weibo during the initial months of the pandemic by analyzing millions of Weibo posts.

Sentiment analysis or opinion mining computationally determines sentiment expressed in text or other media. Lexicon-based approaches are commonly used for sentiment classification due to their effectiveness over supervised approaches [14]. These approaches use a pre-defined dictionary of sentiment-associated terms to determine the sentiment of the text. Researchers have developed sentiment lexicons for this purpose, such as SentiWordNet [15], which ascribes objective, positive, and negative scores to groups of synonymous words. Different approaches exist for applying lexicons, a popular example being VADER (Valence Aware Dictionary and sEntiment Reasoner) [16], a technique intended to mine sentiment in short text fragments like social media posts. Lexicon-based approaches are usually limited to positive or negative sentiment classes and are limited in generalizability compared to transformer-based models; different languages or forms of media would require new lexicons.

On the other hand, traditional machine learning approaches to sentiment analysis, such as Support Vector Machines or Naive Bayes classifiers, have mostly been superseded by transformer-based techniques in recent years due to their strong performance on standard benchmarks [17]. Large language models have also emerged as effective tools for context-rich sentiment analysis [18]. LLMs, including previous-generation models such as Llama 2, have shown promise for English text sarcasm detection [19] and Chinese text sentiment analysis [20]. We use a more modern LLM for non-binary Chinese language sentiment classification in our study.

Sarcasm is a rhetorical device that Merriam-Webster defines as “A sharp and often satirical or ironic utterance designed to cut or give pain.” Sarcasm has been treated as a modifier of positive or negative sentiment that negates or switches the sentiment polarity, as seen in [21,22], which found that switching the naive sentiment polarity of sarcastic texts (positive to negative and vice versa) improved the accuracy of sentiment analysis outcomes. Yet, sarcasm is closely related to irony and satire. According to Giora [23], irony cannot be treated as a mere negation because “irony understanding involves processing both the negated and implicated messages so that analysts may compute the difference between." While the relationship between sarcasm and irony may seem complex, a study of fMRIs [24] found that human brains can differentiate between sarcasm and non-sarcastic irony. Additionally, satire’s definition is “humorous criticism” [25], while sarcasm may serve as the source of humor in satire. Thus, sarcasm can serve many purposes, and some studies treat sarcasm as a binary decision (sarcastic/non-sarcastic), as seen in [26,27]. Our approach to detecting sarcasm as one of four sentiment categories is a flexible alternative to binary detection or negation (see Discussion).

## 2. Materials and Methods

**Figure 1:**
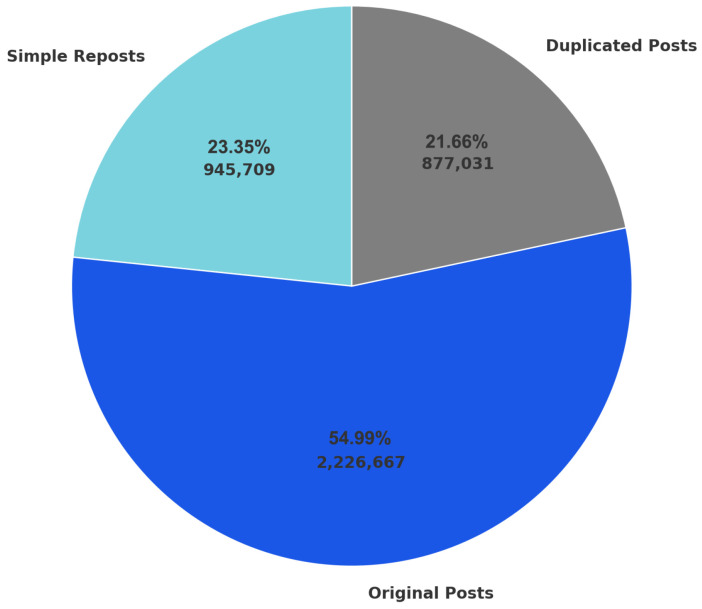
Distribution of duplicate posts, original posts, and reposts. In total, 21.66% of the posts are duplicated (877,031 posts), 54.99% are original posts (2,226,667 posts), and 23.35% of the posts are reposts (945,709 posts).

We use the Weibo COVID-19 Dataset from Harvard [28]. The dataset contains a total of 4,049,407 posts. Based on post content, the dataset includes 2,226,667 distinct posts and 877,031 duplicated posts, or content that may be human-made propaganda or bot-generated spam, and 945,709 reposts. We cleaned and categorized the dataset and presented the types and counts of posts in Figure 1. Some of the duplicates are straightforward to identify as they are entirely identical. However, specific spam posts employ subtle variations, such as adding spaces or tabs, to avoid spam filters on Weibo. To address this issue, we developed a program to preprocess and clean the Weibo dataset, ensuring its quality and consistency before classifying.

We structured the dataset in JSON format. Each post has content and metadata. Table 1 shows this metadata together with their fields.

### 2.1. Post Types and Time Periods

As mentioned earlier, the dataset contains a significant number of duplicate posts. Differentiating between distinct posts, duplicate posts, and reposts is essential to improving classification accuracy.

- Distinct posts: These are original posts created by users with unique content that does not replicate existing posts in the dataset.
- Duplicate posts: These are identical posts that appear multiple times due to content being manually created numerous times without any modifications or automated replication (e.g., spam).
- Reposts: These occur when users share existing posts with or without their comments. Some reposts appear as distinct posts because users have added unique comments or annotations when sharing the content. Conversely, some reposts closely resemble duplicate posts, as they include minimal changes, such as default repost tags or no additional input from the user. Still, we will identify those posts as reposts and exclude them from duplicate posts, as those posts represent individuals’ sentiments just as distinct posts do. We identify reposts by parsing the content of the posts and checking for the presence of “//”. If the post content ends with “//”, it indicates that the post is a repost of another post.

**Figure 2:**
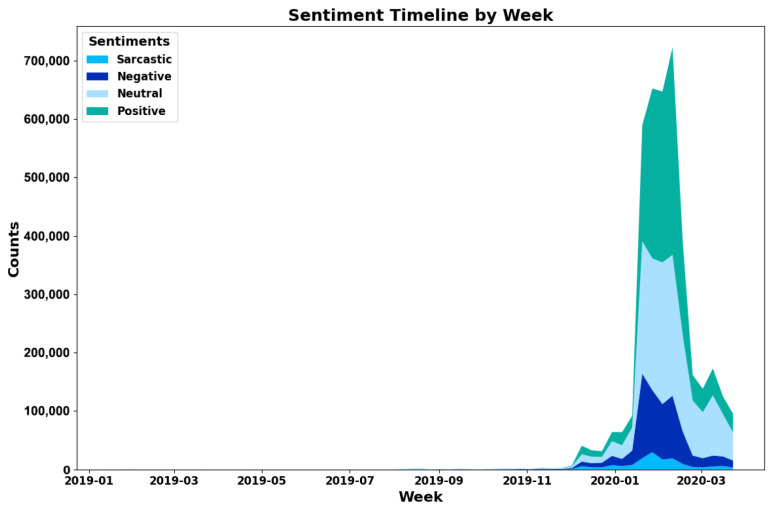
A sentiment timeline stacked area chart illustrating weekly counts of positive, neutral, negative, and sarcastic sentiments from January 2019 to March 2020. Posts in all sentiment categories are significantly stacked around late 2019, peaking in early 2020, with positive and neutral sentiments dominating the counts.

Most posts in the dataset were collected between December 2019 and March 2020, as shown in Figure 2, which also visualizes the posts of each sentiment after running our sentiment classification on the dataset. However, a small number of posts about the African Swine Fever outbreak are also present in the dataset between January 2019 and December 2019.

### 2.2. Few-Shot Prompting

We run the Llama 3 model for sentiment classification of Weibo posts. We use the few-shot prompting technique. We manually identified a few posts containing each of the four sentiment types. Then, we created a prompt for the Llama 3 model to predict the sentiment of posts using five manually labeled posts as an example. We present the prompt we used below. For the convenience of readers not familiar with Chinese, we have translated each post to English using the Google Translate website and placed it under the Chinese version for the readers. These translations were not a part of prompting the model.

“You are a bot designed to judge Chinese sentences as having positive, negative, or neutral sentiment. I’m going to provide posts from Chinese social media. **IMPORTANT: You must only answer with “positive”, “negative”, “sarcastic”, or “neutral”. Do not explain your response or include other text.**

*Use the following criteria for your judgment:*

*1. If the post speaks well of a figure or event commonly regarded as a good figure or event, with no unnecessary exaggeration, it is an example of a ’positive’ sentiment.*

*2. If the post disparages a figure or event commonly regarded as a wrong figure or event, then it is a ’negative’ sentiment.*

*3. If the post does not use emotional language and consists of matter-of-fact reporting of factual statements, it corresponds to a ’neutral’ sentiment.*

*4. Sarcasm in the post, which appears with exaggerated emotion, pretend naivete, or other common sarcastic tone indicators, should correspond to a ’sarcastic’ sentiment.*

*IMPORTANT: Some posts may be appended with the original post that the user replied to. They use the format: [reply]//[original post]. You must predict the sentiment of the reply only, but you may use the original post as context to understand the reply.*

*Here are some examples:*

*Post:* #关注新型肺炎#【国家监委派出调查组，全面调查涉及李文亮医生有关问题】经中央批准，国家监察委员会决定派出调查组赴湖北省武汉市，就群众反映的涉及李文亮医生的有关问题作全面调查。O国家监委派出调查组，全面调查涉及李文亮医生有关问题

*Translation: #focus on new pneumonia# [The National Supervisory Commission dispatched an investigation team to investigate issues related to Dr. Li Wenliang comprehensively]. With the approval of the Central Committee, the National Supervisory Commission decided to send an investigation team to Wuhan City, Hubei Province, to investigate the complaints reported by the masses involving Dr. Li Wenliang’s findings. The National Supervisory Commission dispatched a team of epidemiologists to evaluate these findings.*

*Expected Answer: Neutral*

*Post:* 妈妈我已经快二十天没喝奶茶没有大吃大喝了求求你赶紧疫情结束我人都快没了我想上课我想喝奶茶我想吃烧烤

*Translation: Mom, I have not had milk tea or a big meal for almost 20 days. Please end the epidemic soon. I’m practically dead. I want to go to class, drink milk tea, and have a barbecue.*

*Expected Answer: Negative*

*Post:* 高中时就暗恋他已久，高三毕业那天我鼓起勇气表白，万万没想到他居然也默默喜欢着我，填志愿时也选择了同一个城市。后来工作了异地了四年，晃眼我们走过了十年呢，19年时我们步入了婚姻礼堂。我想这世上最幸福的事情之一那就是两个人都互相深爱并且坚持吧 疫情过后春暖花开，我们想去武大看樱花。

*Translation: I had a crush on them for a long time in high school. On graduation day, I mustered up the courage to confess my love. I never expected that he would secretly like me. When filling out the application form, he chose the same city. Later, I worked in a different place for four years. In the blink of an eye, we have gone through ten years. In 2019, we entered the marriage hall. One of the happiest things in the world is that two people sincerely love each other and persevere. After the epidemic, spring will come, and we want to go to Wuhan University to see the cherry blossoms.*

*Expected Answer: Positive*

*Post:* 就你们敢说实话，//@7362410961:世卫组织说目前只有瑞德西韦可能有效，中科院双黄连有效，南京大学说金银花有效，北京大学沐舒坦有效，南开大学说姜、大枣、龙眼肉都能预防。中国人民真幸福，这么多常见药物、食品可以抗新型冠状病毒，还慌什么呢？

*Translation: You are the only ones who dare to tell the truth, //@7362410961: The World Health Organization said that only Remdesivir may be effective at present, the Chinese Academy of Sciences [stated that] Shuanghuanglian is effective, Nanjing University said that honeysuckle is effective, Peking University Mucosolvan is effective, and Nankai University said that ginger, jujube, and longan meat can prevent it. The Chinese people are so lucky. So many common medicines and foods can resist the new coronavirus. What are they still panicking about?*

*Expected Answer: Sarcastic*

*Post:* 无言//因为疫情我猛然发现不管是新闻里还是现实生活中，从大官到小官，究竟还有多少智力缺陷人士，干出来的事每天都让老百姓瞠目结舌

*Translation: Speechless//Because of the epidemic, I suddenly realized that whether it is in the news or real life, from high officials to minor officials, how many mentally disabled people are there and what they do every day makes people dumbfounded.*

*Expected Answer: Negative*

*Now we begin. Classify:”*

### 2.3. Approach to Sarcasm

We must address our approach to sarcasm detection method. Modern LLMs, such as Llama 3, can understand general language. They are trained with a vast corpus of multi-language tokens from which they gain a general understanding of language, including rhetorical tools such as sarcasm and irony, among many other capabilities. We do not need to align the model with few-shot prompting for it to detect or understand sarcasm. A simpler prompt, such as “Is the following post positive, negative, neutral, or sarcastic?” should also work so the model can distinguish between the four sentiments. However, our approach allows us to prime the model to understand better the context in which these posts exist, the format of the posts, typical examples of various sentiments, and the expected way they must reply. While developing the prompt, we observed that the current version improved inference accuracy for a test set of posts.

In the prompt above, we say that sarcastic posts contain “exaggerated emotion, pretend naivete or other common sarcastic tone indicators”. Such prompt steers the model to the use case. The sarcastic example extends the model by exaggerating a positive tone, sarcastically pointing out the author’s disbelief in alternative COVID-19 cures allegedly suggested by various Chinese institutions. Importantly, this prompt does not teach the model about sarcasm but points it in the right direction for the given use case. The reasoning employed by the model to determine the sentiment of each post is ultimately a black box at this scale. This experiment tests whether the model’s determinations provide an overview of sentiment change on Weibo that human experts can analyze and judge over time.

### 2.4. LLM Accuracy Comparison

We used the instruction-tuned Llama 3 model with 8 billion parameters. We ran the model locally on one of Rensselaer Polytechnic Institute’s FOCI cluster nodes, specifically on an Nvidia Ampere A100 GPU. We downloaded the model from an online platform, HuggingFace, using the HuggingFace Python API. To determine the more suitable model between the 8B parameter versions of Llama 3 and Llama 3.1, we ran a small accuracy comparison. We selected a subset of 199 posts from the dataset to validate the model’s accuracy. We categorized them into positive, negative, neutral, or sarcastic using o1-mini through the OpenAI API. A native Chinese speaker evaluated these labels and found them accurate. Next, we categorized the same subset of posts using our chosen model, Llama 3 8B, and the slightly newer Llama 3.1 8B. Table 2 compares this model’s confusion matrix to the o1-mini model’s classifications, which plays a role of the ground truth.

Llama 3 8B achieves a weighted F1-score of 78.39% overall on the validation set. Llama 3.1 achieves a slightly lower score of 76.88%. Table 3 compares the per-type precision, recall, and F1-score. The table shows that Llamas 3 achieved F1-scores of around 80% for the three well-represented sentiments in the dataset. The exception is sarcastic sentiment, which is the least frequent in the dataset, and its F1-score is much lower at 42.86%. An infrequency of appearance and complexity of expression are the likely reasons for the low F1-score. Llama 3.1 8B is more accurate than Llama 3 for the sarcastic and negative sentiment posts in terms of F1-score, but it is less accurate for the other two types of scores. Therefore, we decided that Llama 3 8B would be the better candidate for our experiment.

### 2.5. Llama 3 8B Alignment Validation

We measure how well-aligned the model is to our specific task with an ’alignment validation’ task. We prepare a suitable validation dataset with ground-truth labels that can test the model’s capability. A few Chinese sentiment datasets are available in the literature, but we found none suitable for our particular task as most have only positive/negative sentiment labels or sarcasm labels but not both. The closest we found is the CMMA benchmark [29], which has separate sentiment and sarcasm annotations. This dataset collects utterances from TV series, and the texts accompany the provided video and audio files to capture the full context, without which sarcasm or sentiment cannot be determined accurately, even by a human speaker. Thus, we had to prepare our dataset.

We used GPT-4o to generate a dataset of 5000 Chinese texts with self-assigned sentiment labels. The GPT-4o model is one of OpenAI’s most powerful models with multi-lingual capabilities. According to OpenAI’s model evaluation of GPT-4o compared to other models [30], it is the best-performing model on benchmarks like MMLU, with stronger performance than Llama 3’s 400B parameter variant. Our choice to generate the dataset instead of annotating a subset of posts from our existing corpus is deliberate. We felt that GPT-4o would assign accurate sentiment labels if it generated its posts and self-assigned its labels consistent with its internal reasoning. This process was also much faster than human annotation. On the other hand, GPT-4o could potentially be less accurate when classifying existing posts as that is a more complex reasoning task, and thus would require closer human oversight. A reasonable use case is using GPT-4o to generate a small labeled validation dataset for the given task.

The generated dataset has 1362 positive texts, 945 negative texts, 1312 neutral texts, and 1381 sarcastic texts. The dataset was generated ten items at a time by calling the OpenAI API with a prompt designed to generate ten texts resembling social media along with ground truth sentiment labels, using the same descriptions of sentiments and examples from the few-shot prompt described in Section 2.2. A native Chinese speaker who looked at a subset of the generated posts confirmed the correctness of the generated posts and their labels. We evaluated the performance of Llama 3 8B on this dataset, and the results are presented in the confusion matrix in Table 4.

The overall accuracy of Llama 3 8B on the validation set is 71.18%, with the F1-scores for the sarcastic, neutral, negative, and positive classes being 61.48%, 73.49%, 69.46%, and 76.41%, respectively. The precision and recall pairs for each class are, respectively, 88% and 47% for sarcastic posts, 63% and 88% for neutral posts, 81% and 61% for negative posts, and 69% and 86% for positive posts. This result illustrates that Llama 3 8B is more well-rounded than the previous accuracy comparison might suggest. While its overall accuracy and the F1-scores for negative, neutral, and sarcastic posts have fallen by a few points, it performs better on sarcastic posts, with the high precision score implying that true sarcastic posts are rarely misclassified. We present further insights from this validation in the Discussion section. In the next section, we detail the outcome of running the model on all posts in the dataset.

## 3. Results

**Figure 3:**
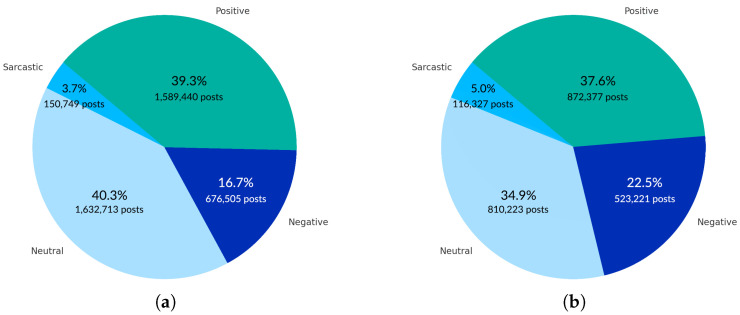
Pie charts show the distribution of posts’ four sentiment categories (positive, neutral, negative, and sarcastic). Chart (**a**) represents the sentiment scale across all posts, while chart (**b**) focuses on distinct (unique) posts with no duplicates.

In the total sentiment distribution, the sentiment of 80% of posts is neutral or positive, as seen in Figure 3a. However, if we discard duplicated posts, the fraction of negative and sarcastic posts increases, and the neutral and positive posts drop by about 10% of the total posts (Figure 3b).

### 3.1. Comparing Trends of COVID-19 and Swine Fever

**Figure 4:**
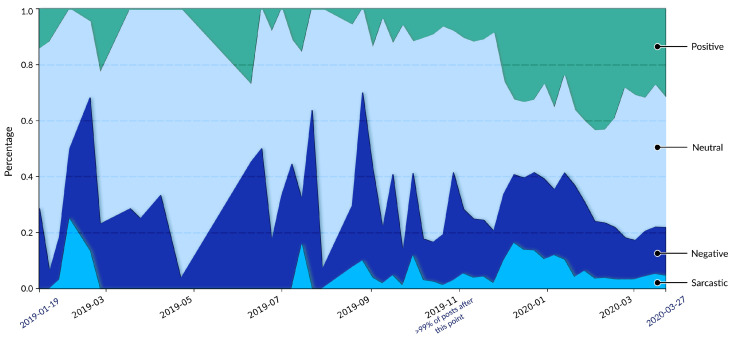
Timeline stacked area chart showing the percentage distribution of four sentiments (positive, neutral, negative, sarcastic) in posts over time. The y-axis represents the current fraction of each sentiment at each time. Each segment’s thickness corresponds to the proportion of that sentiment in the dataset. For example, a wider section indicates a higher percentage of that sentiment during the corresponding period on the x-axis. The x-axis represents days from January 2019 to March 2020, with key events or transitions, such as the note at ‘2019-11’, which marks where over 99% of posts occurred after this point. This chart allows an easy visual comparison of the evolution of each sentiment over time.

Figure 4 shows the sentiment changes for distinct posts over time to reveal further the trends in the evolution of sentiment dynamics. There are two time periods during which there are significant spikes in negative post frequency: one in September 2019 and another in December 2019.

The spike starting in December corresponds to the widely recognized outbreak of COVID-19. Analogically, the spike in negativity around September 2019 is attributable to the outbreak of African Swine Fever in South Korea. Given China’s substantial number of South Korean restaurants and the interconnected food industry between the two countries, this outbreak triggered widespread public concern and panic on Weibo.

While these two harmful spikes were due to disease outbreaks, they exhibit distinct patterns in terms of positive sentiment posts. In the case of September 2019, there is no noticeable increase in positive posts alongside the surge in negative posts. The overall tone on Weibo during this period remains predominantly negative and often sarcastic. In contrast, in December 2019, a substantial rise in positive sentiment posts was observed following the outbreak of COVID-19 in China. Several factors may contribute to these differing patterns. COVID-19 had a more significant impact on daily life in China than African Swine Fever had and fostered greater social cohesion and solidarity than less severe events due to this reason. This cohesion and solidarity led to a more significant presence of positive sentiment posts around the onset of the pandemic. From our analysis, two primary types of posts drove the rise in positive sentiment posts: (1) government efforts to emphasize the effectiveness of disease control measures, including on Weibo, and (2) spontaneous expressions of encouragement and mutual aid among Weibo users.

In contrast, during the September 2019 African Swine Fever outbreak, Weibo made no strong attempt to manage public speech about this disease, and this outbreak’s impact on the daily lives of ordinary citizens in China was minimal. As a result, public discussion was full of rumors, such as concerns about the contamination of supermarket pork. The lack of authoritative communication and reassurance exacerbated public panic, intensifying the negative sentiment on Weibo compared to the outbreak of COVID-19.

### 3.2. Synchronicity of Sentiment Trends

Another notable observation is the synchronicity of dynamics across different sentiments. For instance, sarcastic sentiments and negative sentiments often exhibit synchronized patterns. A rise in sarcastic posts typically accompanies an increase in negative posts. This correlation is understandable, as sarcastic posts frequently convey negative opinions or criticisms.

Interestingly, a correlation occurs between positive and sarcastic sentiments during surges in positive sentiment posts triggered by events. In Figure 4, we can observe an apparent increase in positive, negative, and sarcastic sentiment posts starting in December 2019, as this is the time of the COVID-19 outbreak and the gradual failure of efforts to contain it. A closer examination of sarcastic posts at this point reveals that many are directed at the surge in positive feelings, often satirizing the shift in societal tone. Some sarcastic posts reflect disputes among Weibo users, while others criticize the government.

These debates reached their climax after Dr. Wenliang Li’s death in February 2020. Dr. Li was among the first to identify the new disease outbreak. However, after this report, the government admonished him for spreading rumors and forbade him from discussing COVID-19 further. He later died of COVID-19 infection. After his death, the Chinese government rehabilitated his reputation, acknowledging his contributions to public health and society. We found numerous posts from this period debating the transparency of the Wuhan government (the outbreak’s epicenter), criticizing its inaction, and accusing officials of suppressing information rather than focusing on disease control. As shown in Figure 4, emotions on Weibo rose to a high point in January–Febraury 2020, visualized as neutral posts being compressed into a narrow section. This compression indicates a high level of emotional entropy within the platform during this time. All these internal tensions seem to ease over time. These debates reached their climax after Dr. Wenliang Li’s death in February 2020. Dr. Li was among the first to identify the new disease outbreak. However, after this report, the government admonished him for spreading rumors and forbade him from discussing COVID-19 further. He later died of COVID-19 infection. After his death, the Chinese government rehabilitated his reputation, acknowledging his contributions to public health and society. We found numerous posts from this period debating the transparency of the Wuhan government (the outbreak’s epicenter), criticizing its inaction, and accusing officials of suppressing information rather than focusing on disease control. As shown in Figure 4, emotions on Weibo rose to a high point in January–Febraury 2020, visualized as neutral posts being compressed into a narrow section. This compression indicates a high level of emotional entropy within the platform during this time. All these internal tensions seem to ease over time.

In March 2020, as the pandemic started to spread globally, events such as UK Prime Minister Boris Johnson contracting COVID-19 and U.S. President Donald Trump tweeting praise for China’s response to the epidemic may have contributed to a shift in discourse on Weibo. News about the pandemic in other countries may have become more prominent. As a result, the proportion of negative and sarcastic posts decreased, and neutral sentiment became the dominant voice as news reports were void of emotion.

Our interpretation highlights that real-world events shape societal sentiments and influence one another. A surge in positive sentiments is often met with opposing voices, creating polarization within the online community. Such interactions underscore the complexity of sentiment dynamics in internet societies, where differing perspectives amplify societal divides.

### 3.3. Entropy Analysis of Sentiment Types

We compute the average entropy of posts for each sentiment type. Entropy measures the amount of information content per character, allowing us to compare the complexity of different sentiment categories. For this analysis, we sampled 15,000 posts from each sentiment type and calculated their average entropy in bits. Additionally, we computed the distance between each sentiment type by determining the difference between their entropy, as seen in Table 5. It details the average entropy of each kind and the distances between them. We also paired the neutral and positive sentiments, as they are similar, and calculated their average entropy. Similarly, we paired sarcastic and negative sentiments and provided their average entropy. Lastly, we present the distance between these two pairs.

This novel entropy method allows us to uncover differences between various types of sentiments. Positive and neutral sentiment posts tend to have similar entropy. The reason for this could be that a few unique authors write government, news, or media posts while users reposting them do so without changing much. Such reposting explains the low entropy and thus, lower linguistic diversity of positive and neutral posts. In contrast, negative and sarcastic posts will likely be authored by individuals who have experienced some form of hardship due to recent events. Their expressions of suffering would be personal and unique in this case, leading to a higher entropy due to the limited vocabulary overlapping with other posts.

Sarcastically humorous or satirical language could also explain the higher entropy of sarcastic posts. Satirical texts, for instance, often employ unique word choices that criticize and add humor using the “False analogy” technique [31]. For example, ’BP resumes oil drilling’ is satirized as ‘BP resumes oil spilling’. Sincere posts about a topic would, thus, have lower entropy than satirical posts due to this strategy of replacing words. This is relevant to our finding that sarcasm has high entropy, since satire and sarcasm are closely related.

## 4. Discussion

Sarcasm is often a deliberate attempt at obscuring or exaggerating the deep emotion of the author. Users can post positive or negative emotions earnestly or sarcastically. Thus, using LLMs has one clear advantage over algorithmic or traditional machine learning methods: LLMs understand language by reasoning about the meaning of words in context instead of relying on a dictionary of phrases or the limitations of just the training datasets. The ideal LLM should be able to perform text opinion mining at scale, with its outcomes representing one subjective but acceptable interpretation of the sentiment of each text. Our work is a methodical step toward this ultimate goal.

Sentiment mining of text is inherently subjective, as it represents an attempt to read into the author’s intent. Social media posts are typically short and information sparse, limiting space for emotional weight. Thus, in a social network, users express sarcasm via text posts by careful phrasing or rely on commonly understood sarcastic expressions. Poorly phrased sarcasm can be misunderstood by other users as earnestness or humor. To avoid such misunderstanding, our prompt to Llama 3 8B instructs it to detect sarcasm via ’commonly understood’ forms of sarcastic phrasing, for which it depends on its training and its reasoning ability.

Our non-binary classification system combines multi-modal and binary classification. Consider a hypothetical multi-modal classification in which posts have a sentiment classification (positive/negative/neutral) and a binary sarcasm flag (a sarcastic flag is labeled one while a non-sarcastic flag is labeled zero). In this case, a post with a positive sentiment, which is also sarcastic, has at least two interpretations. The first interpretation is that the positive sentiment is sarcastic, while the author’s intent is negative, which means sarcasm played the role of a polarity switch used in [21,22]. The other interpretation is that the author’s actual intent is positive, and they used sarcasm to modify a negative phrase, in which case it would not be correct to switch the sentiment polarity. In addition, there may also be cases where sarcastic posts have neutral sentiments. Our non-binary classification treats sarcasm as a separate sentiment category. The sarcastic posts in our system can be categorized as either positive or negative in future research, depending on their rhetorical purpose (satire, irony, or humor), which will define their polarization.

Our alignment validation for Llama 3 8B in Section 2.5 reveals a pattern where positive and neutral classifications have low precision and high recall compared to negative and sarcastic classifications, which have the opposite problem. The model’s low precision for positive and neutral posts is caused by frequent mistakes of assigning a positive label to a neutral post and vice versa, as shown in Table 4. Our entropy analysis found that neutral and positive posts have similar and lower entropy scores than the other two types. This outcome confirms a similarity between positive and neutral posts, which may be one possible explanation for why the model has difficulty separating positive and neutral classes. Other work on LLM-based sentiment analysis has highlighted this weakness as well [32]. Similarly, we found that negative and sarcastic posts are close in entropy levels but higher than those for the other two sentiment types. Their higher entropy levels indicate they contain more unique terms than the other pair. This difference between pairs explains why the model finds the two classes separable, resulting in a high precision score for both. The low recall score for these two types shows that the model is reluctant to classify a post as negative or sarcastic but has a high degree of accuracy when it does. These properties may reflect a bias from its training data and a need to fine-tune the model better.

We use entropy for comparing and characterizing the content of social media posts based on the diversity or similarity of the language used by the authors of the posts. In [33], the authors defined the conversational trust of two users, *A* and *B*, denoted as $T_{c}\left(A,B\right)$, and defined as follows: $T_{c}\left(A,B\right)=\sum_{i=1}^{\tau }\left|\right|C_{i}\left|\right|H\left(C_{i}\right)$, where $H(C_{i})$ is a measure of the balance in the conversation. They used the entropy function to measure this balance: $H\left(C_{i}\right)=-p\log p-\left(1-p\right)\log\left(1-p\right)$, where $p(C_{i})$ is the fraction of messages in the conversation $C_{i}$ that were sent by *A*. Then, the authors verify that frequent, long, and balanced conversations yield a high value of $T_{c}$ and indicate trust between interacting users. In conclusion, the authors wrote: “Trust is an important yet complex and little understood aspect of the dyadic relationship between two entities. Trust plays an important role in forming coalitions in social networks and determining how the high value of information flows through the network. The authors present algorithmically quantifiable measures of Trust based on communication behavior”. The authors conjecture that trust results in similar behaviors that are statistically different from random communications.

In [34], the authors observe that: “Human communication is commonly represented as a temporal social network, and evaluated in terms of its uniqueness”. The authors propose new entropy-based measures for human communication dynamics represented within the temporal social network as event sequences. The authors used real-world datasets and different types of random interaction series to find patterns of actual human contact events. They devised a metric that always significantly distinguishes human patterns from random ones.

In [35], the authors focused on online social media that provided many open-ended platforms for users of various backgrounds, interests, ages, education, and beliefs to interact and debate. This platform attracted many users, who engaged in countless discussions across a wide range of subjects. These diverse and unique voices spread to the ever-growing information streams. It is fundamental to consider how the types of conversations that result from a social media post interpret the posts themselves. The authors hypothesized that: “the biases and predispositions of users cause them to react to different topics in different ways not necessarily entirely intended by the sender”. The authors then introduced unique features that capture discourse patterns, allowing them to empirically explore the relationship between a topic and the conversations it induces. Utilizing “microscopic” trends to describe “macroscopic” phenomena, the authors set a paradigm for analyzing information dissemination through the user reactions triggered by a topic, eliminating the need to investigate the text in the discussions. Using a Reddit dataset, the authors found that the selected features not only enable classifiers to distinguish between content genres accurately but can also identify more subtle semantic differences in content under a single topic and isolate outliers whose subject matter is substantially different from the norm.

In all three cases above, the entropy metric has the advantage of relying on patterns of posts and the diversity of vocabularies used by individuals in their posts that can be analyzed without understanding the words. In our case of sentiment, we take advantage of the fact that professionals organized by the government often prepare the most positive posts. Those are well-educated, mature professionals who write posts inspired by their superiors. As a result, the posts use very similar vocabularies. Thus, the words differing from the first post are small; we will call the authors of professional posts dependent on each other and measure them by overlapping their words in posts. Our paper shows that users of many positive posts replicate them, causing slow growth of unique words with the development of the number of authors and lowering the entropy of positive posts. Negative and sarcastic posts written by the same number of independent, diverse authors with different ages and professions who live in diverse places will have a smaller overlap of words. Importantly, the writer of negative posts suffers from the crisis, according to the quote from Tolstoy’s novel ’Anna Karenina’, reading “All happy families are alike; each unhappy family is unhappy in its unique way”. This quote applies to our scenario, since government writers are unlikely to suffer from the crisis while negative post-writers do, and they will use new words to describe their unique suffering. By doing so, suffering writers increase the number of unique words in their posts, thereby increasing their post entropy.

In future work, we will determine how often the crisis scenario happens. We will also attempt to study fake news and conspiracy posts inspired by [36], in which the authors study metrics for discovering fake news, which is not a new problem, and its spread in social networks is well-studied. Yet, the authors wrote that they conducted: “...a unique study of three datasets and features that capture the style and the language of articles, showing that fake news in most cases is more similar to satire than to real news, leading us to conclude that fake news posts achieve their persuasion through heuristics rather than the strength of arguments”. The authors also showed that titles’ title structure and proper nouns significantly differentiate fake from real.

While the model’s accuracy is limited, the alignment validation enables us to interpret the possible distortion in the outcome. Extrapolating based on the per-class precisions and recalls, one alternate interpretation is that the actual proportion of neutral posts is lower, the proportions of negative and sarcastic posts is higher, and positive posts are slightly lower. However, the pattern of sentiments seen follows observable trends and has entropy characterizations that make sense, and thus, the picture we get is useful.

The timeline of sentiments illustrated by classifications of Llama 3 8B shown in Figure 4 is one interpretation of sentiment evolution on Weibo during the COVID-19 pandemic. This interpretation is useful for officials, agencies, and social media companies, as it shows the organically emerging pattern of sentiment on social media, allowing these institutions to understand events and the sentiments they spur among the public. This approach has important limitations, which we discuss in the Conclusion. The model may also be biased, as its classifications carry the model’s training and reasoning biases. For example, bias towards certain public figures or events and bias toward marginalized groups may be present. It is important that user-facing applications of this method that would affect people, such as using this technique in content filtration or moderation, consider if the model has a sentiment bias toward such groups or figures. Researchers and engineers should ensure that LLM-based techniques are not used to censor ideas or sentiments. Such censorship may prove detrimental to public trust in the platform over time.

## 5. Conclusions

### 5.1. Contributions

Our study presents two novel contributions to sentiment analysis. First, we created a large-scale sentiment classification of approximately three million Chinese-language posts from the social network Weibo using the Llama 3 8B model. A few-shot learning prompt with manually verified examples to guide the model was evaluated using a generated set of Chinese posts with self-assigned labels. Following dataset cleaning, we started on 2,322,148 original posts and categorized them, leading to a final classification of 4,049,407 posts, including duplicates.

Second, we analyzed sentiment trends during the early stages of the COVID-19 pandemic from November 2019 to March 2020. The sentiment expressed in online discussions regarding COVID-19 was more favorable than discussions of the African Swine Fever outbreak a few months earlier. Additionally, we identify a correlation between the frequency of sarcastic posts and fluctuations in sentiment polarity. The management of COVID-19 influenced the trends by discourse and the polarization of opinions on Weibo.

This study demonstrates the effectiveness of large language models in large-scale sentiment analysis of Chinese-language posts. Traditional NLP methods often struggle to capture rhetorical devices, grammatical nuances, and informal language that influence sentiment classification, particularly in non-English languages. Using large language models addresses these challenges by enabling context-aware sentiment classification and improving sarcasm detection.

### 5.2. Limitations

Llama 3 8B has decent F1-scores and per-class precision and recall values, but there is much room for improvement. Typical opinion mining methods sometimes do not include sarcasm and even neutrality, so this approach is more sophisticated at the cost of accuracy. Even though the model’s accuracy is limited, it was still effective when identifying patterns and trends of the Weibo sentiments as a whole. Further improvements in sarcasm detection accuracy are necessary for future large-scale sentiment analyses, particularly for capturing complex linguistic patterns exhibited by users. A more sophisticated LLM such as GPT-4o or Llama 3 400B would almost certainly improve the outcome. Still, the computing resources and costs associated with deploying such large models at scale are a limiting factor. A good compromise would be fine-tuning a smaller model with a labeled dataset.

The synthetic dataset used for alignment validation in Section 2.5 has self-assigned labels GPT-4o determined as it generated the posts. Although the correctness of these labels was checked by a native speaker for a subset of generated posts and found to be highly accurate, this method should be used carefully, as self-assigned labels are not traditional ground-truth labels and require human oversight. Also, the number of posts used for this validation is a small fraction of the total number of posts in the target dataset. We imposed this limitation due to the paucity of suitable datasets with suitable ground-truth labels in the literature and the large time and resource investment required to build a dataset of adequate size. While our validation outcomes are still useful, there is a risk that we did not trigger negative aspects of the model.

Our methods are generalizable to other languages supported by Llama 3, although it is important to conduct a literature review to estimate the model’s effectiveness for a particular language. In addition, our methods would be useful for sentiment classification of datasets containing short, context-rich text fragments such as other social media, literary quotes, and similar data. Long text classification would be more compute-intensive, and therefore, less scalable. Modelers should not exceed the context window of smaller LLMs. Yet, as new LLMs are released over time, better alternatives to Llama 3 8B will likely emerge for scalable opinion mining.

### 5.3. Future Works

Detection of rhetorical elements in sentiment classification using large language models is still an open area for further investigation. Future research should explore the impact of different rhetorical devices, not only sarcasm, in social networks. Another important aspect for future studies is interpretability and reasoning mechanisms employed by large language models when making sentiment classifications. Our work also highlights the need for further work on the relationship between entropy and sentiment of text, including how LLM-based sentiment analysis may be affected by entropy variation.

## Figures and Tables

**Table 1:** Metadata of each post in the dataset.

Field Name	Field Description
From	Device used to post
Content	Text content of the post
Repost—Content	Text content of the repost
Repost—Imgs	Images in the repost
Repost—Timestamp	Timestamp of the repost
Repost—Username	Numeric ID identifying the repost
Timestamp	Timestamp of the post
User_ID	Numeric ID identifying the poster
Weibo_ID	Unique ID of the post

**Table 2:** Llama 3 and o1-Mini Confusion Matrix. The diagonal highlighted in green shows the number of accurate classifications for each class.

	Sarcastic	Neutral	Negative	Positive
Sarcastic	3	3	1	0
Neutral	1	69	1	5
Negative	1	8	25	1
Positive	2	17	3	59

**Table 3:** Comparison of precision, recall, and F1-scores of Llama 3 and 3.1.

	Llama 3			Llama 3.1		
**Metric>**	**Precision**	**Recall**	**F1-Score**	**Precision**	**Recall**	**F1-Score**
Sarcastic	0.4286	0.4286	0.4286	0.5714	0.4444	0.5000
Neutral	0.7113	0.9079	0.7977	0.7320	0.8353	0.7802
Negative	0.8333	0.7143	0.7692	0.9000	0.7105	0.7941
Positive	0.9077	0.7284	0.8082	0.7846	0.7612	0.7727

**Table 4:** Llama 3 Confusion Matrix on GPT-4o self-assigned dataset. The diagonal highlighted in green shows the number of accurate classifications for each class.

	Sarcastic	Neutral	Negative	Positive
Sarcastic	652	214	120	395
Neutral	1	1159	10	142
Negative	87	283	572	3
Positive	0	186	0	1176

**Table 5:** Sentiment entropy and distance.

Type	Entropy	Rank	Distance	Pair Entropy	Pair Distance
Neutral	9.2740	Lowest	0.0559	9.30195	0.1306
Positive	9.3299	Low	0.0785		
Sarcastic	9.4084	High	0.0483	9.43255	
Negative	9.4567	Highest	-		

## Data Availability

The data presented in this study are available in the ’Weibo COVID dataset’ repository on the Harvard Dataverse available at DOI 10.7910/DVN/DULFFJ. The raw data supporting the conclusions of this article (sentiment classifications) will be made available by the authors on request.
